# Development and validation of genic-SSR markers in sesame by RNA-seq

**DOI:** 10.1186/1471-2164-13-316

**Published:** 2012-07-16

**Authors:** Haiyang Zhang, Libin Wei, Hongmei Miao, Tide Zhang, Cuiying Wang

**Affiliations:** 1 Henan Sesame Research Center, Henan Academy of Agricultural Sciences, Zhengzhou, 450002, Henan, P. R. China

## Abstract

**Background:**

Sesame (*Sesamum indicum* L*.*) is one of the most important oil crops; however, a lack of useful molecular markers hinders current genetic research. We performed transcriptome sequencing of samples from different sesame growth and developmental stages, and mining of genic-SSR markers to identify valuable markers for sesame molecular genetics research.

**Results:**

In this study, 75 bp and 100 bp paired-end RNA-seq was used to sequence 24 cDNA libraries, and 42,566 uni-transcripts were assembled from more than 260 million filtered reads. The total length of uni-transcript sequences was 47.99 Mb, and 7,324 SSRs (SSRs ≥15 bp) and 4,440 SSRs (SSRs ≥18 bp) were identified. On average, there was one genic-SSR per 6.55 kb (SSRs ≥15 bp) or 10.81 kb (SSRs ≥18 bp). Among perfect SSRs (≥18 bp), di-nucleotide motifs (48.01%) were the most abundant, followed by tri- (20.96%), hexa- (25.37%), penta- (2.97%), tetra- (2.12%), and mono-nucleotides (0.57%). The top four motif repeats were (AG/CT)n [1,268 (34.51%)], (CA/TG)n [281 (7.65%)], (AT/AT)n [215 (5.85%)], and (GAA/TTC)n [131 (3.57%)]. A total of 2,164 SSR primer pairs were identified in the 4,440 SSR-containing sequences (≥18 bp), and 300 SSR primer pairs were randomly chosen for validation. These SSR markers were amplified and validated in 25 sesame accessions (24 cultivated accessions, one wild species). 276 (92.0%) primer pairs yielded PCR amplification products in 24 cultivars. Thirty two primer pairs (11.59%) exhibited polymorphisms. Moreover, 203 primer pairs (67.67%) yielded PCR amplicons in the wild accession and 167 (60.51%) were polymorphic between species. A UPGMA dendrogram based on genetic similarity coefficients showed that the correlation between genotype and geographical source was low and that the genetic basis of sesame in China is narrow, as previously reported. The 32 polymorphic primer pairs were validated using an F_2_ mapping population; 18 primer pairs exhibited polymorphisms between the parents, and 14 genic-SSRs could be integrated into 9 main linkage groups.

**Conclusions:**

2,164 genic-SSR markers have been developed in sesame using transcriptome sequencing. 276 of 300 validated primer pairs successfully yielded PCR amplicons in 24 cultivated sesame accessions. These markers increase current SSR marker resources and will greatly benefit genetic diversity, qualitative and quantitative trait mapping and marker-assisted selection studies in sesame.

## Background

Sesame (*Sesamum indicum* L., 2n = 26), belonging to the *Pedaliaceae* genus, is an ancient oilseed crop, considered important for its high quality seed oil [1]. Sesame is cultivated mainly in the tropical and subtropical regions of Asia and Africa, with a total area of 7.7 million hectares worldwide and an annual production of 3.98 million tons (2009, FAO data, http://faostat.fao.org/site/567/DesktopDefault.aspx?PageID=567). In China, one of the main long-term hindrances in sesame production is the lack of varieties with high disease resistance and water-logging tolerance. Genetic diversity among cultivars is relatively low since all varieties are derived from the one cultivated sesame species, *Sesamum indicum* L. The low level of polymorphism in sesame has been demonstrated using universal markers such as random amplified polymorphic DNA (RAPD) [2,3], inter-simple sequence repeats (ISSR) [4], amplified fragment length polymorphism (AFLP) [5] and sequence-related amplified polymorphisms (SRAP) [6], and species-specific markers such as simple sequence repeats (SSR) [7] and expressed sequence tags-SSR (EST-SSR) [8]. Inadequate information on sesame resistance to biotic and abiotic stresses, and sesame growth and developmental processes has created a breeding bottleneck which is unlikely to be solved in the near future.

Since massive-scale cloning and sequencing of DNA or EST libraries has been relatively high-cost, low throughput and time-consuming, the development of SSR markers has been slow, making it more difficult to construct a detailed genetic linkage map that can be used in sesame genetics breeding programs. At present, including a recently published set of 40 sesame SSR markers derived from a transcriptome study [7-9], less than 80 polymorphic SSR and EST-SSR markers are available. At present, only eight EST-SSR markers are anchored in the first and only sesame genetic map [10].

Recent advances in large-scale RNA-seq provide a fast, cost-effective, and reliable approach for the generation of large expression datasets in non-model species [11-13], and also offer an opportunity to identify and develop SSRs using data mining with bioinformatic tools. Compared with genomic SSR markers, these new genic-SSR markers may help to identify candidate functional genes and increase the efficiency of marker-assisted selection [14]. We therefore performed sesame RNA-seq to further our understanding of the sesame transcriptome and to develop large numbers of novel and efficient genic-SSR molecular markers. Here, we analyze the frequency and distribution of genic-SSRs in the sesame RNA-seq transcriptome, and validate 300 of our 2,164 SSR markers in 24 cultivated accessions, one wild species and one F_2_ mapping population. Our set of SSR markers will provide a useful tool for sesame genetic research and comparative genome analysis.

## Results

### Uni-transcript sequences obtained with Illumina sequencing

We obtained more than 260 million 75 bp or 100 bp paired-end filtered reads from 24 sesame samples using high-throughput paired-end RNA-seq. The total length of the reads was over 45.85 Gbp. Reads were subsequently *de novo* assembled into 342,776 contigs with a length of over 100 bp, and then further assembled into 42,566 uni-scaffolds with a mean size of 1,127 bp using paired-end joining and TGI Clustering tools (Table 1).

**Table 1:** Transcriptome statistics

**Contig length**	**Number**	**Percentage (%)**	**Uni-scaffold length**	**Number**	**Percentage (%)**
100 ~ 200 bp	205,735	60.02	100 ~ 200 bp	4,613	10.84
201 ~ 300 bp	70,767	20.65	201 ~ 300 bp	5,727	13.45
301 ~ 400 bp	26,685	7.79	301 ~ 400 bp	3,786	8.89
401 ~ 500 bp	14,143	4.13	401 ~ 500 bp	2,709	6.36
501 ~ 600 bp	8,174	2.38	501 ~ 600 bp	2,053	4.82
601 ~ 700 bp	5,052	1.47	601 ~ 700 bp	1,756	4.13
701 ~ 800 bp	3,336	0.97	701 ~ 800 bp	1,534	3.60
801 ~ 900 bp	2,332	0.68	801 ~ 900 bp	1,415	3.32
901 ~ 1000 bp	1,514	0.44	901 ~ 1000 bp	1,343	3.16
1001 ~ 2000 bp	4,567	1.33	1001 ~ 2000 bp	10,256	24.09
2001 ~ 3000 bp	422	0.12	2001 ~ 3000 bp	4,616	10.84
3001 ~ 10 kbp	49	0.01	3001 ~ 10 kbp	2,734	6.42
>10 kbp	0	0.00	>10 kbp	24	0.06
Total Contigs	342,776	100.00	Total Uni-scaffolds	42,566	100.00
Total Length (bp)	82,262,551		Total Length (bp)	47,986,977	
N50 Length (bp)	263		N50 Length (bp)	1,901	
Mean Length (bp)	239		Mean Length (bp)	1,127	

### Mining of genic-SSRs

The 42,566 uni-transcript sequences covered 47,987 kbp of the sesame genome, and a total of 7,324 (≥15 bp) and 4,440 (≥18 bp) SSRs, present in 17.21% and 10.43% of the uni-transcripts respectively, were identified in the data.

### Types and frequencies of genic-SSRs

We divided the SSRs into three groups according to the repeat motif classification criteria proposed by Weber [15], i.e., perfect, imperfect and compound types (Table 2). Most repeats (SSRs ≥15 bp: 6,485, 88.54%; SSRs ≥18 bp: 3,674, 82.75%) were perfect repeats. Of these, di-nucleotide repeats were the most abundant motif type.

**Table 2:** Repeat motif type distribution in ≥15 bp and ≥18 bp genic-SSRs

**Repeat motif type**			**SSRs ≥15 bp**		**SSRs ≥18 bp**	
			**Number**	**Frequency (%)**	**Number**	**Frequency (%)**
Perfect	Mono-		129	1.99	21	0.57
	Di-		2,592	39.97	1,764	48.01
	Tri-		1,845	28.45	770	20.96
	Tetra-		335	5.17	78	2.12
	Penta-		652	10.05	109	2.97
	Hexa-		932	14.37	932	25.37
	Total		6,485	100.00	3,674	100.00
Imperfect	Mono-		82	37.27	77	38.31
	Di-		137	62.27	123	61.19
	Tri-		1	0.45	1	0.50
	Total		220	100.00	201	100.00
Compound	Perfect	Mono-Mono-	35	14.29	22	9.78
		Di-Di-	199	81.22	193	85.78
		Tri-Tri-	4	1.63	4	1.78
		Mono-Di-	4	1.63	3	1.33
		Mono-Tri-	2	0.82	2	0.89
		Di-Tri-	1	0.41	1	0.44
		Total	245	100.00	225	100.00
	Imperfect	Mono-Mono-	12	3.21	12	3.53
		Di-Di-	352	94.12	318	93.53
		Tri-Tri-	6	1.60	6	1.76
		Mono-Di -	1	0.27	1	0.29
		Mono-Tri-	1	0.27	1	0.29
		Di-Tri-	2	0.53	2	0.59
		Total	374	100.00	340	100.00
Total			7,324		4,440	

In the imperfect and compound SSR categories, only mono-, di- and tri-nucleotide SSR units were present. All repeat motifs in mono-nucleotide SSR units were of the A/T type. AG/CT, CA/TG and AT/AT repeat motif types were present in di-nucleotide SSR units, while only GAA/TTC repeat motifs were found in tri-nucleotide SSR units. Of the six types of SSR units, mono-mono, di-di-, tri-tri-, mono-di-, mono-tri- and di-tri-nucleotide types were found in both perfect and imperfect compound SSR categories. The di-di-nucleotide type was the most abundant, representing more than 80% of all SSRs.

### Distribution of repeat motif types

**Figure 1:**
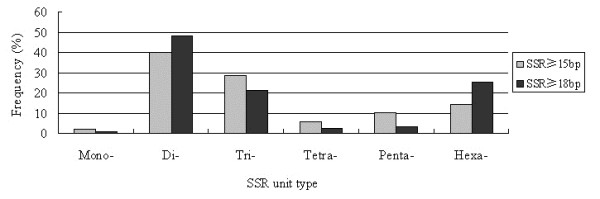
Frequency distribution of the six perfect SSR unit types.

We noted that the proportion of six different SSR unit sizes was not evenly distributed among perfect SSR groups. Different repeat units occurred at frequencies of: 1.99% and 0.57% (mono-nucleotides) 39.97% and 48.01% (di-nucleotides), 28.45% and 20.96% (tri-nucleotides), 5.17% and 2.12% (tetra-nucleotides), 10.05% and 2.97% (penta-nucleotides), and 14.37% and 25.37% (hexa-nucleotides), for SSRs ≥15 bp and ≥18 bp, respectively (Figure 1).

A total of 687 and 557 types of repeat motifs were identified among the 6,485 (SSRs ≥15 bp) and 3,674 (SSRs ≥18 bp) perfect SSRs (Table 3). The (A/T)n mono-nucleotide repeat motif was the most abundant in both datasets. The five other main unit types were the (AG/CT)n di-nucleotide, (GAA/TTC)n tri-nucleotide, (ATAC/GTAT)n tetra-nucleotide, (AAAAG/CTTTT)n penta-nucleotide and (GAAAAA/TTTTTC)n hexa-nucleotide repeat motifs, and occurred at frequencies of 98.45% and 100%, 66.86% and 71.88%, 15.12% and 17.01%, 10.15% and 17.95%, 8.59% and 3.67%, and 2.36% and 2.36%, in SSRs ≥15 bp and SSRs ≥18 bp, respectively. Furthermore, it was observed that the G/C repeat motif type was only present in mono-nucleotide SSR units in SSRs ≥15 bp; and the GC/GC repeat motif type was not observed in di-nucleotide SSR units in either SSRs ≥15 bp or SSRs ≥18 bp.

**Table 3:** Number and frequency of six types of perfect SSR repeat motif in sesame

**SSR motif unit**	**Repeat motif number and frequency**		**Most abundant type**
	**SSR ≥15 bp**	**SSR ≥18 bp**	
Mono-	2 (0.29%)	1 (0.18%)	(A/T)n
Di-	3 (0.44%)	3 (0.54%)	(AG/CT)n
Tri-	18 (2.62%)	18 (3.23%)	(GAA/TTC)n
Tetra-	50 (7.28%)	33 (5.92%)	(ATAC/GTAT)n
Penta-	184 (26.78%)	72 (12.93%)	(AAAAG/CTTTT)n
Hexa-	430 (62.59%)	430 (77.20%)	(GAAAAA/TTTTTC)n
Total	687 (100%)	557 (100%)	

Of the perfect motif types, the (AG/CT)n di-nucleotides were the most abundant (SSRs ≥15 bp, 1,733 (26.72%); SSRs ≥18 bp, 1,268 (34.51%)), followed by (CA/TG)n di-nucleotides (469 (7.23%) and 281 (7.65%)), (AT/AT)n di-nucleotides (390 (6.01%) and 215 (5.85%)), and (GAA/TTC)n tri-nucleotides (279 (4.3%) and 131 (3.57%)).

Further analysis indicated that the copy number of different repeat motifs in perfect SSRs sequences was distributed unevenly (Table 4). The copy number of different repeat motifs varied from 3 to 26, with the (AG/CT)n di-nucleotide repeats having the highest copy number. The four most frequent copy numbers for SSRs ≥15 bp were 3 (19.81%), 5 (18.13%), 8 (14.09%) and 9 (9.16%), while 3 (20.20%), 9 (16.17%), 6 (12.82%) and 10 (10.13%) were the most frequent copy numbers for SSRs ≥18 bp. The longest SSR length in each unit type (from mono- to hexa- nucleotide repeats) was 25 bp (A/T), 52 bp (AG/CT), 51 bp (GAA/TTC and TGA/TCA), 32 bp (TATG/CATA and TACA/TGTA), 55 bp (ATTCC/GGAAT) and 48 bp (TGATGG/CCATCA).

**Table 4:** Frequency of different repeat motifs in perfect SSRs (≥15 bp and ≥18 bp)

**Number of Motif copies**	**Mono-**	**Di-**	**Tri-**	**Tetra-**	**Penta-**	**Hexa-**	**Total**	**Frequency (%)**
2	0	0	0	0	0	0	0	0.00 (0.00)
3	0	0	0	0	543	742	1,285 (742)	19.81 (20.20)
4	0	0	0	257	93	150	500 (243)	7.71 (6.61)
5	0	0	1,075	59	10	32	1,176 (101)	18.13 (2.75)
6	0	0	452	14	1	4	471	7.26 (12.82)
7	0	0	169	3	1	3	176	2.71 (4.79)
8	0	828	81	2	2	1	914 (86)	14.09 (2.34)
9	0	549	45	0	0	0	594	9.16 (16.17)
10	0	358	13	0	1	0	372	5.74 (10.13)
11	0	254	4	0	1	0	259	3.99 (7.05)
12	0	178	2	0	0	0	180	2.78 (4.90)
13	0	103	1	0	0	0	104	1.60 (2.83)
14	0	70	0	0	0	0	70	1.08 (1.91)
15	51	50	0	0	0	0	101 (50)	1.56 (1.36)
16	40	52	1	0	0	0	93 (53)	1.43 (1.44)
17	17	28	2	0	0	0	47 (30)	0.72 (0.82)
18	11	19	0	0	0	0	30	0.46 (0.82)
19	4	7	0	0	0	0	11	0.17 (0.30)
20	2	16	0	0	0	0	18	0.28 (0.49)
21	0	10	0	0	0	0	10	0.15 (0.27)
22	1	19	0	0	0	0	20	0.31 (0.54)
23	0	23	0	0	0	0	23	0.35 (0.63)
24	2	21	0	0	0	0	23	0.35 (0.63)
25	1	5	0	0	0	0	6	0.09 (0.16)
26	0	2	0	0	0	0	2	0.03 (0.05)
Total	129	2,592	1,845	335	652	932	6,485	100.00
	(21)	(1,764)	(770)	(78)	(109)	(932)	(3,674)	
Frequency (%)	1.99	39.97	28.45	5.17	10.05	14.37	100.00	
	(0.57)	(48.01)	(20.96)	(2.12)	(2.97)	(25.37)		

Data for SSRs ≥18 bp is given in brackets.

### PCR amplification and polymorphism of genic-SSRs

**Figure 2:**
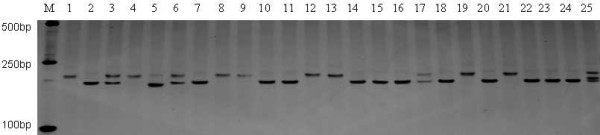
**Polymorphism of the primer HS233 in 25 sesame accessions.** 6% PAGE of 24 cultivar accessions and one wild species M: DNA marker; Lanes 1 ~ 25: Samples M1 ~ 25 (Additional file 2).

Using Primer3, 2,164 SSR primer pairs were detected in the 4,440 SSR-containing sequences (SSR ≥18 bp) and 300 SSR primer pairs were randomly selected and synthesized to validate their level of polymorphism (Additional file 1: Table S1). Of these primer pairs, 7 (2.33%) amplified non-specific products, and 17 (5.67%) gave no products in any of the sesame accessions. 276 (92.0%) primer pairs yielded amplification products in the 24 cultivars, of which 32 (11.59%) exhibited polymorphisms. A total of 74 alleles were detected with these 32 primer pairs and the number of alleles ranged from 2–4 per genic-SSR marker, with a mean of 2.31. As shown in Figure 2, the HS233 SSR marker detected the maximum number of alleles (4). 203 (67.67%) of the SSR primer pairs yielded PCR amplicons in the wild accession, 167 (60.51%) of which were polymorphic between the wild accession and cultivated accessions.

### Phylogenetic analysis of the 24 cultivated sesame accessions

**Figure 3:**
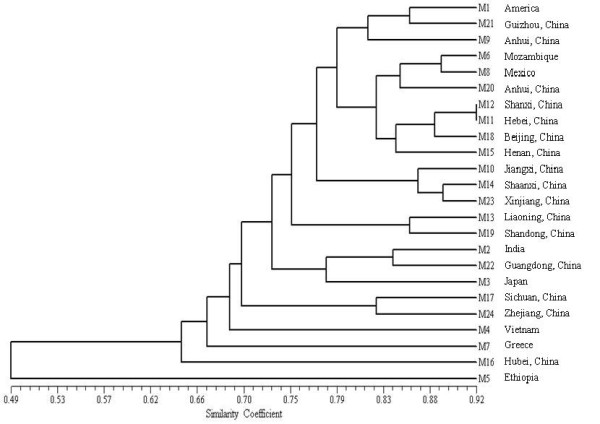
**UPGMA dendrogram of the genetic relationships among 24 cultivated sesame accessions.** The dendrogram was generated using the Jaccard similarity coefficient based on 32 polymorphic primer pairs.

In order to evaluate their ability to assess molecular diversity and their potential for use in fingerprinting analysis, we calculated the PIC values of the above genic-SSR markers, based on the allelic variation exhibited by 32 polymorphic primer pairs in 24 cultivated accessions. PIC values ranged from 0.08 to 0.67, and had an average value of 0.34 (Additional file 1: Table S1), with primer HS233 giving the maximum PIC value of 0.67. Phylogenetic relationships between the cultivars were assessed by constructing a UPGMA dendrogram using similarity coefficients (Figure 3). At a similarity coefficient ≥ 0.75, the largest subgroup consisted of 15 accessions, comprising 7 Chinese-released cultivars, 5 Chinese local sesame accessions and 3 exotic sesame accessions. The M5 accession (Gonder-2) had the lowest similarity value of 0.49 and was clustered into a distant subgroup. The next most distant cultivars were M16 and M7, splitting into subgroups at similarity values of 0.66 and 0.64, respectively. Our results indicate that geographic sources of the accessions in this study do not correspond well with the genetic distances between accessions and as a result the genetic relationships among exotic, local germplasm and cultivars are not clear.

### Genetic mapping

**Figure 4:**
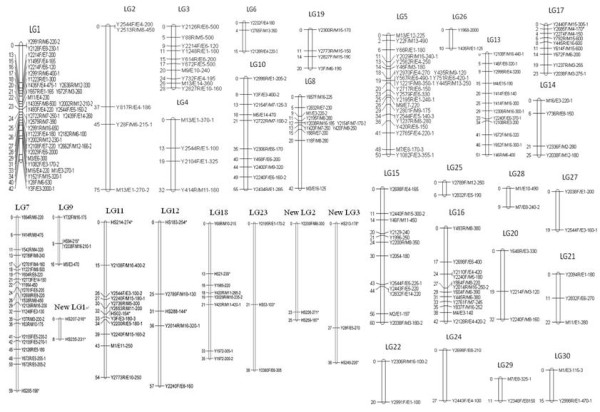
**Distribution of 14 new polymorphic SSR markers across the 9 linkage groups of the F2 backbone genetic linkage map.** * new sesame genic-SSR markers.

The analysis above indicated that 18 markers (6.52%) were polymorphic between the parents of our mapping population (M16 and M17). After screening the 96 F_2_ mapping population, 14 genic-SSR markers were distributed among 9 linkage groups (Figure 4).

## Discussion

In order to identify useful SSR markers and obtain transcriptomic information on disease resistance and developmental processes, we sequenced the transcriptomes of 24 sesame samples and identified 2,164 genic-SSR primer pairs (SSRs ≥18 bp).

Genic-SSR markers are considered to have strong potential for genetic analysis and linkage map construction in crop species due to their specificity and high degree of conservation [16-21]. Although 120 EST-SSRs have previously been developed from 3,428 EST sequences and utilized in sesame genetic diversity analysis and mapping [8,10,22], polymorphic markers are few, and marker-assisted gene mapping for important sesame traits or biological processes such as disease resistance, sesame growth and development, and seed formation has thus not been widely implemented.

### Genic-SSR distribution

Here, to accurately analyze the frequency of SSRs in the transcribed regions of the sesame genome, we compared the numbers and types of SSR motif sequences of SSRs ≥15 bp and ≥18 bp. A total of 7,324 (17.21%) (SSRs ≥15 bp) and 4,440 SSRs (10.43%) (SSRs ≥18 bp) were identified in 42,566 uni-transcript sequences, with an average of one SSR per 6.55 kb and 10.81 kb, respectively. By the parameter of sequence length (Kb) per SSR marker, the distribution frequency of genic SSRs is both lower than that of previous EST-SSRs developed from EST sequences in sesame (8.68% (SSRs ≥18 bp), one EST-SSR per 4.99 kb) [8]. This frequency of occurrence of sesame genic-SSRs (SSRs ≥18 bp) is relatively higher than in other crops, including wheat (one EST-SSR per 17.42 kb), rice (one per 11.81 kb), maize (one per 28.32 kb) and soybean (one per 23.80 kb) [23]. Furthermore, it has been emphasized that the frequency of SSRs is correlated with many factors, such as SSR detection criteria, dataset size, database-mining tools, different species and different materials [8,24].

### Distribution of repeat motif types

Of the perfect repeat motifs types, tri-nucleotide repeats have generally been observed to have the highest frequency in many crops, including cotton, barley, wheat, maize, sorghum, rice and peanut [25-27]. However, here, as in previous studies on sesame and some *Rosaceae* species, the most abundant repeat motif type was the di-nucleotide [8,28]. Hexa-nucleotide repeats were the second most abundant (25.37%), followed by tri-nucleotides (20.96%) in SSRs ≥18 bp. Moreover, of the hundreds of types of repeat motifs, the (AG/CT)n di-nucleotide motifs showed the highest frequency, in agreement with recent results in sesame and other species [8,27,29,30]. As in other dicot plants, such as *Arabidopsis*[29], soybean [23] and peanut [26], but different from some cereal species [27,31,32], the (GAA/TTC)n motif was the most abundant of the tri-nucleotide repeat motifs. Similar to wheat, sorghum and peanut [26,27], the GC/GC repeat was not found in any of the perfect and imperfect SSR categories in sesame.

### Polymorphic nature of the genic-SSR markers

To determine the level of polymorphism among our set of new genic-SSR markers, we validated 300 primer pairs using 25 sesame accessions. 276 (92.0%) successfully yielded PCR amplicons, in line with previously reported ratios of 60–92.2% amplification [8,23,28,33-36]. 203 (73.55%) of the genic-SSRs that yielded amplifiable products in cultivated sesame also produced PCR amplicons in a wild sesame species. The ratio of polymorphic SSR was similar to that for EST-SSRs in other crops with a range of 40–89% [16,17,31,37,38].

Some reports indicated that the low polymorphism of SSR markers in sesame is likely due to its narrow genetic basis [7,8]. Dixit et al. (2005) found that only ten out of 50 SSR markers developed from a sesame DNA library were polymorphic in 16 sesame accessions [7]. Wei et al. (2008) developed 50 EST-SSR markers from the 3,328 sesame ESTs published in NCBI, and found that only 27 (61.4%) were polymorphic in the 36 sesame accessions tested (34 cultivated sesame accessions and 2 wild sesame accessions) [8]. In this study, a similar level of polymorphism was observed; only 32 (11.59%) genic-SSR markers were polymorphic in 24 cultivars, 18 (6.52%) were polymorphic in one mapping population, and 167 (60.51%) were polymorphic between the 24 acessions and a wild sesame accession. Furthermore, the level of polymorphism in sesame was also similar to other crops [21,26]. In wheat, no more than 6.25% of primers exhibit polymorphisms between the parents of any individual mapping population, although 81.25% of detected EST-SSRs have been reported to exhibit polymorphisms in 18 alien species [21]. In peanut, 26 (10.3%) EST-SSRs exhibited polymorphisms between 22 cultivated peanut accessions and 221 (88%) were polymorphic between 16 wild peanut species [26].

Our results indicate that large numbers of polymorphic SSR markers can be obtained when large volumes of transcript sequences or datasets are used, even though genetic diversity is restricted in sesame cultivars. Compared with other SSR detection methods, the *de novo* RNA sequencing approach used here is well-suited for mining and developing large numbers of genic-SSRs in sesame, and can rapidly enrich the numbers of functional markers available to use in marker-assisted gene selection and QTL analysis.

### Phylogenetic analysis of 24 cultivated sesame accessions

Our dendrogram, based on genetic similarity results, did not divide our sesame accessions into clear groupings. The distribution of these sesame accessions was not based on their geographical sources, in agreement with some previous reports [2-5]. The average PIC value of genic-SSRs obtained here was 0.34, similar to that obtained in our previous study [8]. Most of the varieties released in China were clustered in the same subgroup in the dendrogram, suggesting the limited genetic diversity and narrow basis of Chinese sesame cultivars. To enlarge the genetic basis, more exotic accessions should be used in future sesame breeding programs. One possibility would be to introduce Gonder-2 (M5, Ethiopia), the outlying accession in our dendrogram, as a parent for sesame breeding or other genetic research.

### Utilization of genic-SSR markers in genetic mapping

We anchored 14 of our newly developed genic-SSR markers in the sesame genetic map (Figure 4), nearly twice the number of those anchored in recent sesame genetic map study [10]. Using these newly designed genic-SSRs, the density of SSR markers in the sesame genetic map will greatly increase in the near future. In addition, putative functions of 11 of the 14 anchored genic-SSRs were identified with BLASTX. These genic-SSRs will be very valuable in studies of gene mapping, comparative genome analysis and marker-assisted selection.

## Conclusions

2,164 genic-SSR markers were identified from 42,566 uni-scaffolds in a comprehensive transcriptome study. 276 of the 300 primer pairs chosen for validation successfully yielded PCR amplicons in 24 cultivated sesame accessions. This set of genic-SSR markers will be valuable for genetic research in sesame on aspects such as growth and development processes or biotic stress traits, since our transcriptome data was derived from different organs, developmental stages, and stress treatments.

## Methods

### Plant materials

The 24 samples analysed in RNA-seq experiments (Additional file 2: Table S2), included four accessions of cultivated sesame (*Sesamum indicum* L., 2n = 26), one wild species (*Sesamum radiatum* Schum. & Thonn., 2n = 64) and their distant hybrid progeny. Samples were grown under normal conditions in a greenhouse at 25°C with 14 h light per day, or in an experimental field at Yuanyang Experimental station, HAAS. To evaluate biotic stress, seedlings were inoculated with a 10^6^/mL conidiophore suspension of *Fusarium oxysporum* f. sp. *sesami* (No. HSFO 09030) for 0, 6, 24 or 48 h at 25°C in a greenhouse before harvesting. Control plants were inoculated with sterilized water. Plant parts, including the whole seedling, developing seeds (harvested at different days after flowering (DAF)), germinated seeds, and developing flowers (1-8 mm size), were harvested, immersed in liquid nitrogen and stored at -70°C before RNA extraction.

The 24 cultivated accessions and one wild species used (Additional file 3: Table S3) to validate the polymorphic nature of genic-SSR candidate markers were samples from the sesame germplasm collection at the Henan Sesame Center, HAAS, Zhengzhou, China. The F_2_ segregating population used to validate the 300 sesame genic-SSR marker candidates consisted of 96 lines and was the same as that used in the construction of the first sesame genetic map [10].

### RNA isolation and library preparation

Total RNA was isolated with TRIzol (Invitrogen) according to the manufacturer’s instructions and total mRNA was then purified using oligo (dT) magnetic beads. cDNA libraries were prepared according to Illumina sequencing sample preparation protocols. In total, 24 paired-end cDNA libraries were constructed with an insert size ranging from 280 bp to 320 bp.

### Illumina sequencing and *de novo* transcriptome assembly

cDNA libraries were sequenced on an Illumina sequencing platform (GAII) using a 75 bp or 100 bp paired-end approach. Integrated high-quality paired-end Illumina reads (>Q20) were assembled using the *de novo* assembler Velvet and Oases [39]. After all adaptor sequences, empty reads and low quality sequences were removed from the raw reads, the resultant contigs were built into uni-scaffolds based on paired-end information using TGI Clustering (TGICL) tools [40].

### SSR detection and development of primer pairs

To detect SSR markers, 42,566 uni-transcript sequences containing 2-6 repeat motifs were screened using SSRIT [41], and mono-nucleotide SSRs were identified using its EditPlus function. The SSR motif detection criterion was a minimum length of either 15 or 18 bases. Primers for the ≥18 bp genic-SSRs in microsatellite sequences were designed with Primer3 [42], based on the following core criteria: a G/C content between 40% and 70%, an annealing temperature between 54°C and 63°C, a minimum product length of 100 bp, and a primer length of 18–24 nucleotides. All candidate SSR primer pairs were synthesized by BGI (Shenzhen, China). Functional analysis of the transcriptome sequences was carried out with blastn and blastx (NCBI).

### DNA extraction, PCR amplification and electrophoresis

To validate the SSR markers, genomic DNA was extracted from 25 accessions as described by Paterson et al. [43]. DNA amplification was performed in a 10 μL reaction mixture containing 1 × Buffer, 2.0 mmol/L MgCl_2_, 0.1 mmol/L dNTPs, 1 μmol/L of each primer, 0.5 U *Taq* polymerase, and 80 ng template DNA. SSR-PCR amplification was performed on a PTC-225 machine (MJ Research, MA, USA) using the following profile: 1 cycle of 3 min at 94°C, 31 cycles of 1 min at 94°C, 50 s at 56–63°C, 1 min at 72°C and a final cycle of 6 min at 72°C. Amplicon electrophoresis was performed as described by Zhang et al. [44].

### SSR genetic similarity analysis and mapping

To estimate the allelic variation of SSRs in the 25 accessions, the polymorphism information content (PIC) of each SSR primer was calculated as following: *PIC =* 1-$\sum_{i=1}^{n}$*Pi*^*2*^, where *Pi* is the frequency of the *i*^th^ allele for a given SSR marker, and *n* is the total number of alleles detected for that SSR marker [45]. Coefficients of genetic similarity for the 24 cultivated accessions used in this study were calculated using the SIMQUAL program of NTSYS-pc Version 2.10 [46]. A neighbor-joining dendrogram was constructed based on the genetic similarity matrix with the SHAN clustering program [33,47] of NTSYS-pc using the UPGMA algorithm. We used 18 of our new polymorphic markers to screen the 96 F_2_ segregation population, 14 of which were integrated into the first sesame genetic linkage map using JoinMap ver. 3.0 program [48].

## Competing interests

The authors declare that they have no competing interests.

## Authors’ contributions

ZHY designed the study and finalized the manuscript. WLB carried out the SSR mining and validating experiment and drafted the manuscript. MHM coordinated the study, prepared the materials for transcriptome sequencing and performed the transcriptome information analysis. ZHT and WCY screened the SSR markers and mapping. Transcriptome sequencing and assembly was outsourced to Illumina, China. All authors read and approved the final manuscript.

## Supplementary Material

Additional file 1 **Characteristics of sesame genic-SSR primers used in this study.** The SSR primer name, primer sequence, annealing temperature, repeat motif, product length, allele no., PIC value, E-value (nr) and annotation (nr) are given. Click here for file

Additional file 2 24 sesame samples used for RNA-seq. Click here for file

Additional file 3 **Characteristics of the 25 sesame accessions used in the SSR validation.** M1 ~ M8 are exotic sesame accessions from 8 countries; M9 ~ M16 are China released sesame cultivars, M17 ~ M24 are China local sesame accessions, M25 is a wild species (*Sesamum radiatum*). (DOC 51 kb) Click here for file

## References

[B1] Ashri A Sesame breeding Plant Breeding Review 1998 16 179 228

[B2] Bhat KV Babrekar PP Lakhanpaul S Study of genetic diversity in Indian and exotic sesame (Sesamum indicum L.) germplasm using random amplified polymorphic DNA (RAPD) markers Euphytical 1999 110 21 33

[B3] Ercan AG Taskin M Turgut K Analysis of genetic diversity in Turkish sesame (Sesamum indicum L.) populations using RAPD markers Genet Res Crop Evol 2004 51

[B4] Kim DH Zur G Danin-Poleg Y Lee S Shim K Kang C Kashi Y Genetic relationships of sesame germplasm collection as revealed by inter-simple sequence repeats Plant Breed 2002 121 259 262

[B5] Hernan EL Petr K Genetic relationship and diversity in a sesame (Sesamum indicum L.) germplasm collection using amplified fragment length polymorphism (AFLP) BMC Genet 2006 7 10 16483380 10.1186/1471-2156-7-10 PMC1434769

[B6] Zhang YX Zhang XR Hua W Wang LH Che Z Analysis of genetic diversity among indigenous landraces from sesame (Sesamum indicum L.) core collection in China as revealed by SRAP and SSR maikers Genes & Genomics 2010 32 207 215

[B7] Dixit AA Jin MH Chung JW Yu JW Chung HK Ma KH Park YJ Cho EG Development of polymorphic microsatellite markers in sesame (Sesamum indicum L.) Mol Ecol Notes 2005 5 736 738

[B8] Wei LB Zhang HY Zheng YZ Guo WZ Zhang TZ Developing EST-derived microsatellites in sesame (Sesamum indicum L.) Acta Agron Sin 2008 34 12 2077 2084

[B9] Wei WL Qi XQ Wang LH Zhang YX Hua W Li DH Lv HX Zhang XR Characterization of the sesame (Sesamum indicum L.) global transcriptome using Illumina paired-end sequencing and development of EST-SSR markers BMC Genomics 2011 12 451 21929789 10.1186/1471-2164-12-451 PMC3184296

[B10] Wei LB Zhang HY Zheng YZ Miao HM Zhang TZ Guo WZ A Genetic linkage map construction for sesame (Sesamum indicum L.) Genes & Genomics 2009 31 2 199 208

[B11] Marioni J Mason C Mane S Stephens M Gilad Y RNA-seq: An assessment of technical reproducibility and comparison with gene expression arrays Genome Res 2008 18 1509 1517 18550803 10.1101/gr.079558.108 PMC2527709

[B12] Nagalakshmi U Wang Z Waern K Shou C Raha D Gerstein M Snyder M The transcriptional landscape of the yeast genome defined by RNA sequencing Science 2008 320 1344 1349 18451266 10.1126/science.1158441 PMC2951732

[B13] Mortazavi A Williams BA Williams BA Mccue K Schaeffer L Mapping and quantifying mammalian transcriptomes by RNA-Seq Nat Methods 2008 5 7 621 628 18516045 10.1038/nmeth.1226 PMC13303166

[B14] Gupta PK Rustgi S Molecular markers from the transcribed/expressed region of the genome in higher plants Funct Integr Genomics 2004 4 3 139 162 15095058 10.1007/s10142-004-0107-0

[B15] Weber JL Informativeness of human (dC-dA)n·(dG-dT)n polymorphisms Genomics 1990 7 524 530 1974878 10.1016/0888-7543(90)90195-z

[B16] Yu JK LaRota M Kantety RV Sorrells ME EST derived SSR markers for comparative mapping in wheat and rice Mol Gen Genet 2004 271 742 751 10.1007/s00438-004-1027-3 15197579

[B17] Varshney RK Sigmund R Borner A Korzun V Stein N Sorrells ME Langridge P Graner A Interspecific transferability and comparative mapping of barley EST-SSR markers in wheat, rye and rice Plant Sci 2005 168 195 202

[B18] Xie WG Zhang XQ Cai HW Liu W Peng Y Genetic diversity analysis and transferability of cereal EST-SSR markers to orchardgrass (Dactylis glomerata L) Biochemical systematics and ecology 2010 38 4 740

[B19] Cordeiro GM Casu R McIntyre CL Manners JM Henry RJ Microsatellite markers from sugarcane (Saccharum spp) EST cross transferable to erianthus and sorghum Plant Sci 2001 160 1115 1123 11337068 10.1016/s0168-9452(01)00365-x

[B20] Saha MC Rouf Mian MA Eujayl I John CZ Wang LJ May GD Tall fescue EST-SSR markers with transferability across several grass species Theor Appl Genet 2004 109 783 791 15205737 10.1007/s00122-004-1681-1

[B21] Gupta PK Rustgi S Sharma S Singh R Kumar N Balyan HS Transferable EST-SSR markers for the study of polymorphism and genetic diversity in bread wheat Mol Gen Genet 2003 270 315 323 10.1007/s00438-003-0921-4 14508680

[B22] Suh MC Kim MJ Hur CG Bae JM Park YI Chung CH Kang CW Ohlrogge JB Comparative analysis of expressed sequence tags from Sesamum indicum and Arabidopsis thaliana developing seeds Plant Mol Biol 2003 52 6 1107 1123 14682612 10.1023/b:plan.0000004304.22770.e9

[B23] Gao LF Tang JF Li HW Analysis of microsatellites in major crops assessed by computational and experimental approaches Mol Breed 2003 12 245 261

[B24] Varshney RK Graner A Sorrells ME Genic microsatellite markers in plants: features and applications Trends Biotechnol 2005 23 1 48 55 15629858 10.1016/j.tibtech.2004.11.005

[B25] Wang CB Guo WZ Cai CP Characterization, development and exploitation of EST-derived microsatellites in Gossypium raimondii Ulbrich Chin Sci Bull 2006 51 316 320

[B26] Liang XQ Chen XP Hong YB Liu HY Zhou GY Li SX Guo BZ Utility of EST-derived SSR in cultivated peanut (Arachis hypogaea L.) and Arachis wild species BMC Plant Biol 2009 9 35 19309524 10.1186/1471-2229-9-35 PMC2678122

[B27] Kantety RV Rota ML Matthews DE Data mining for simple sequence repeats in expressed sequence tags from barley, maize, rice, sorghum and wheat Plant Mol Biol 2002 48 501 510 11999831 10.1023/a:1014875206165

[B28] Sook J Abbott A Jesudurai C Frequency, type, distribution and annotation of simple sequence repeats in Rosaceae EST Funct Integr Genom 2005 5 136 143 10.1007/s10142-005-0139-0 15761705

[B29] Cardle L Ramsay L Milbourne D Computational and experimental characterization of physically clustered simple sequence repeats in plants Genetics 2000 156 847 854 11014830 10.1093/genetics/156.2.847 PMC1461288

[B30] Jia XP Shi YS Song YC Development of EST-SSR in foxtail millet (Setaria italica) Genet Resour Crop Evol 2007 54 233 236

[B31] Peng JH Lapitan NL Characterization of EST-derived microsatellites in the wheat genome and development of eSSR markers Funct Integr Genomics 2005 5 80 96 15650880 10.1007/s10142-004-0128-8

[B32] Thiel T Michalek W Varshney RK Graner A Exploiting EST databases for the development and characterization of genederived SSR-markers in barley (Hordeum vulgare L.) Theor Appl Genet 2003 106 3 411 422 12589540 10.1007/s00122-002-1031-0

[B33] La Rota M Kantety RV Yu JK Sorrells ME Nonrandom distribution and frequencies of genomic and EST-derived microsatellite markers in rice, wheat, and barley BMC Genomics 2005 6 23 15720707 10.1186/1471-2164-6-23 PMC550658

[B34] Xin Y Cui HR Zhang ML Development of EST (expressed sequence tags) Marker in Chinese cabbage and its transferability to rapeseed Hereditas (Beijing) 2005 27 410 416 15985406

[B35] Chabane K Ablett GA Cordeiro GM EST versus genomic derived microsatellite markers for genotyping wild and cultivated barley Genet Resour Crop Evol 2005 52 903 909

[B36] Cloutier S Niu Z Datla R Duguid S Development and analysis of EST-SSRs for flax (Linum usitatissimum L.) Theor Appl Genet 2009 119 53 63 19357828 10.1007/s00122-009-1016-3

[B37] Nicot N Chiquet V Gandon B Amilhat L Legeai F Leroy P Bernard M Sourdille P Study of simple sequence repeat (SSR) markers from wheat expressed sequence tags (ESTs) Theor Appl Genet 2004 109 800 805 15146317 10.1007/s00122-004-1685-x

[B38] Yu JK Dake TM Singh S Benscher D Li W Gill B Sorrells ME Development and mapping of EST-derived simple sequence repeat markers for hexaploid wheat Genome 2004 47 5 805 818 15499395 10.1139/g04-057

[B39] Zerbino DR Birney E Velvet: algorithms for de novo short read assembly using de Bruijn graphs Genome Research 18 821 829 18349386 10.1101/gr.074492.107 PMC2336801

[B40] Pertea G Huang X Liang F Antonescu V Sultana R Karamycheva S Lee Y White J Cheung F Parvizi B TIGR Gene Indices clustering tools (TGICL): a software system for fast clustering of large EST datasets Bioinformatics 2003 19 651 652 12651724 10.1093/bioinformatics/btg034

[B41] Temnykh S DeClerck G Lukashova A Computational and experimental analysis of microsatellites in rice (Oryza sativa L.): frequency, length variation, transposon associations, and genetic marker potential Genome Research 2001 11 8 1441 1452 11483586 10.1101/gr.184001 PMC311097

[B42] Rozen S Skaletsky HJ Krawetz S, Misener S Primer3 on the www for general users and for biologist programmers Bioinformatics Methods and Protocols: Methods in Molecular Biology 2000 Humana Press, Totowa, NJ 365 386 10.1385/1-59259-192-2:365 10547847

[B43] Paterson AH Brubaker C Wendel JF A rapid method for extraction of cotton (Gossypium spp) genomic DNA suitable for RFLP or PCR analysis Plant Mol Biol 1999 11 122 127

[B44] Zhang J Wu YT Guo WZ Fast screening of microsatellite markers in cotton with PAGE/silver staining Acta GossypiiSin 2000 12 267 269 in Chinese with English abstract

[B45] Park YH Alabady MS Ulloa M Genetic mapping of new cotton fiber loci using EST-derived microsatellites in an interspecific recombinant inbred (RIL) cotton population Mol Genet Genom 2005 274 428 441 10.1007/s00438-005-0037-0 16187061

[B46] Rohlf FJ NTSYS-pc: Numerical Taxonomy and Multivariate Analysis System, Version 2.1 2000 Exeter Software, New York

[B47] Sneath PH Sokal RR Numerical Taxonomy: The Principal and Practice of Numerical Classification 1973 W. H. Freeman and Company, San Francisco

[B48] Van Ooijen JW Voorrips RE JoinMap 3.0, Software for the calculation of genetic linkage maps 2001 Plant Research International Wageningen, The Netherlands

