# Supplementary material for: Development and validation of genic-SSR markers in sesame by RNA-seq
Source: BMC Genomics. 2012 Jul 16;13:316. doi: 10.1186/1471-2164-13-316 (PMC3428654; doi:10.1186/1471-2164-13-316)
Supplement: Additional file 2 — 24 sesame samples used for RNA-seq. [file 1471-2164-13-316-S2.doc]

Additional File 2: 24 sesame samples used for RNA-seq

| **Materials** | **Stages** | **Organs** | **Treatments** |
| --- | --- | --- | --- |
| YZ11 | 3-39 DAF | developing seed | none |
| YZ11 | flowering | anther | none |
| YZ11 | 30 d | seedling | none |
| YZ11 | 30 d | seedling | inoculated for 6 h with *Fusarium oxysporum* f.sp. *sesami* |
| YZ11 | 30 d | seedling | inoculated for 24 h with *Fusarium oxysporum* f.sp. *sesami* |
| YZ11 | 30 d | seedling | inoculated for 48 h with *Fusarium oxysporum* f.sp. *sesami* |
| YZ11 | 48-96 h | germinating seed | none |
| YZ11 | 48 h | germinating seed | inoculated for 6 h with *Fusarium oxysporum* f.sp. *sesami* |
| YZ11 | 48 h | germinating seed | inoculated for 24 h with *Fusarium oxysporum* f.sp. *sesami* |
| YZ11 | 48 h | germinating seed | inoculated for 48 h with *Fusarium oxysporum* f.sp. *sesami* |
| RXBS | 30 d | seedling | none |
| RXBS | 30 d | seedling | inoculated for 6 h with *Fusarium oxysporum* f.sp. *sesami* |
| RXBS | 30 d | seedling | inoculated for 24 h with *Fusarium oxysporum* f.sp. *sesami* |
| RXBS | 30 d | seedling | inoculated for 48 h with *Fusarium oxysporum* f.sp. *sesami* |
| RXBS | 48-96 h | germinating seed | none |
| RXBS | 48 h | germinating seed | Inoculated for 6 h with *Fusarium oxysporum* f.sp. *sesami* |
| RXBS | 48 h | germinating seed | inoculated for 24 h with *Fusarium oxysporum* f.sp. *sesami* |
| RXBS | 48 h | germinating seed | inoculated for 48 h with *Fusarium oxysporum* f.sp. *sesami* |
| Wild sesame 1* | 30 d | seedling | none |
| F1 hybrid** | 30 d | seedling | none |
| COI1134 | 30 d | seedling | none |
| ms86-1 | flowering | fertile anther | none |
| ms86-1 | flowering | sterile anther | none |
| ms86-1 | flowering | partial sterile anther | none |

*: Wild sesame 1 (*Sesamum radiatum*)

**: Parents: YZ4 tetraploid and Wild sesame 1 (*Sesamum radiatum*)
