# Supplementary material for: Development and validation of genic-SSR markers in sesame by RNA-seq
Source: BMC Genomics. 2012 Jul 16;13:316. doi: 10.1186/1471-2164-13-316 (PMC3428654; doi:10.1186/1471-2164-13-316)
Supplement: Additional file 3 — Characteristics of the 25 sesame accessions used in the SSR validation. M1 ~ M8 are exotic sesame accessions from 8 countries; M9 ~ M16 are China released sesame cultivars, M17 ~ M24 are China local sesame accessions, M25 is a wild species (Sesamum radiatum). (DOC 51 kb) [file 1471-2164-13-316-S3.doc]

**Additional File : Characteristics of 25 sesame accessions provided for SSR validation**

| **No.** | **Accession name** | **Sources** | **Domestication** |
| --- | --- | --- | --- |
| M1 | CR205SH | America | Exotic cultivar |
| M2 | PbieNO.1 | India | Exotic cultivar |
| M3 | Japan sesame 435 | Japan | Exotic cultivar |
| M4 | Huang sesame | Vietnam | Exotic cultivar |
| M5 | Gonder-2 | Ethiopia | Exotic cultivar |
| M6 | Margo(01311-B)-1 | Mozambique | Exotic cultivar |
| M7 | Proimo | Greece | Exotic cultivar |
| M8 | Ciano37 | Mexico | Exotic cultivar |
| M9 | Wanzhi 1 | Anhui, China | Cultivar |
| M10 | Ganzhi 6 | Jiangxi, China | Cultivar |
| M11 | Jizhi 1 | Hebei, China | Cultivar |
| M12 | Jinzhi 1 | Shanxi, China | Cultivar |
| M13 | Liaozhi 1 | Liaoning, China | Cultivar |
| M14 | Shaanzhi 1 | Shaanxi, China | Cultivar |
| M15 | Yuzhi 11 | Henan, China | Cultivar |
| M16 | China oil institute 1134 | Hubei, China | Cultivar |
| M17 | Rongxian black sesame | Sichuan, China | Germplasm |
| M18 | Daxing | Beijing, China | Germplasm |
| M19 | Luodihuang | Shandong, China | Germplasm |
| M20 | Silencao | Anhui, China | Germplasm |
| M21 | Qingong sesame | Guizhou, China | Germplasm |
| M22 | Jianzui sesame | Guangdong, China | Germplasm |
| M23 | Emin white sesame | Xinjiang, China | Germplasm |
| M24 | Ningbo black sesame | Zhejiang, China | Germplasm |
| M25 | Wild sesame 1 | India | Wild germplasm |

Note: No. M1~M8 are exotic sesame accessions from 8 countries; M9~M16 are China released sesame cultivars, M17~M24 are China local sesame accessions, M25 is a wild species (*Sesamum radiatum*).
